# Health-related quality of life dynamics: modeling insights from immunotherapy

**DOI:** 10.1007/s11136-024-03810-0

**Published:** 2024-10-30

**Authors:** Zeynep Hasgul, Anne Spanjaart, Sumreen Javed, Ali Akhavan, Marie José Kersten, Mohammad S. Jalali

**Affiliations:** 1 MGH Institute for Technology Assessment, Harvard Medical School, 125 Nashua St, Boston, MA 02114 USA; 2 Department of Hematology, Amsterdam UMC location University of Amsterdam, Amsterdam, The Netherlands; 3 Cancer Center Amsterdam, Amsterdam, The Netherlands; 4 LYMMCARE (Lymphoma and Myeloma Center Amsterdam), Amsterdam, The Netherlands; 5 Sloan School of Management, Massachusetts Institute of Technology, Cambridge, MA USA

**Keywords:** Health-related quality of life, Over-time dynamics, Simulation modeling, CAR T-cell therapy, Cancer immunotherapy

## Abstract

**Background:**

Understanding health-related quality of life (HRQoL) dynamics is essential for assessing and improving treatment experiences; however, clinical and observational studies struggle to capture their full complexity. We use simulation modeling and the case of Chimeric Antigen Receptor T-cell therapy—a type of cancer immunotherapy that can prolong survival, but carries life-threatening risks—to study HRQoL dynamics.

**Methods:**

We developed an exploratory system dynamics model with mathematical equations and parameter values informed by literature and expert insights. We refined its feedback structure and evaluated its dynamic behavior through iterative interviews. Model simulated HRQoL from treatment approval through six months post-infusion. Two strategies—reducing the delay to infusion and enhancing social support—were incorporated into the model. To dynamically evaluate the effect of these strategies, we developed four metrics: post-treatment HRQoL decline, recovery time to pre-treatment HRQoL, post-treatment HRQoL peak, and durability of the peak.

**Results:**

Model captures key interactions within HRQoL, providing a nuanced analysis of its continuous temporal dynamics, particularly physical well-being, psychological well-being, tumor burden, receipt and efficacy of treatment, side effects, and their management. Model analysis shows reducing infusion delays enhanced HRQoL across all four metrics. While enhanced social support improved the first three metrics for patients who received treatment, it did not change durability of the peak.

**Conclusions:**

Simulation modeling can help explore the effects of strategies on HRQoL while also demonstrating the dynamic interactions between its key components, offering a powerful tool to investigate aspects of HRQoL that are difficult to assess in real-world settings.

**Supplementary Information:**

The online version contains supplementary material available at 10.1007/s11136-024-03810-0.

## Abstract (plain language summary)

Understanding how treatments affect patients’ quality of life over time is crucial, but capturing the complex interactions of health factors poses a challenge for clinical and observational research. To overcome this, we have turned to simulation modeling, a method that allows for a more thorough exploration of these dynamics. Our study focuses on cancer immunotherapy, a treatment that, despite its potential to prolong survival, also comes with life-threatening risks. We evaluated the effectiveness of two strategies aimed at improving quality of life: reducing the time to treatment infusion and enhancing social support. These strategies were assessed across three different patient scenarios: those not initially eligible for treatment, patients experiencing a relapse, and patients showing a complete response. By using simulation modeling, we demonstrated how this approach can help explore the dynamics and interactions of various health factors and the impact of specific strategies.

**Supplementary Information:**

The online version contains supplementary material available at 10.1007/s11136-024-03810-0.

## Background

Understanding the dynamics of health-related quality of life (HRQoL) is essential for comprehending the overall treatment experience of patients. HRQoL encompasses multiple dimensions of well-being, reflecting patient’s subjective assessment of their physical, psychological, social, and functional status as influenced by their health condition and its treatment. Given the interactions between these HRQoL dimensions, understanding and improving HRQoL can be challenging [1]. Moreover, studying HRQoL through clinical and observational methods is costly due to complicated or infeasible data collection across multiple variables, rigorous ethical oversight, and the need for resources and specialized personnel [2]. Additionally, data collection over short time intervals (e.g., daily, weekly) is not always feasible, and it is challenging to collect data over the long term. In contrast, simulation modeling can provide a powerful tool to overcome these challenges and explore hypotheses in a computer-based setting, exploring ‘what-if’ questions that may not be feasible to analyze in the real world [3, 4]. We, therefore, built a simulation model to study the dynamics of HRQoL, using CAR (Chimeric Antigen Receptor) T-cell therapy as a case study.

Chimeric Antigen Receptor (CAR) T-cell therapy is a type of immunotherapy that enhances patient’s immune system to fight cancer by using genetically engineered T-cells designed to target tumor antigens. CAR T-cell therapy has revolutionized cancer care, showing efficacy, especially against B-cell lymphomas [5–8], acute lymphoblastic leukemia [5], and multiple myeloma [9]. It offers a treatment option for patients with advanced-stage cancer who have relapsed or not responded to other treatments [10, 11] and has demonstrated the potential to achieve remissions lasting over five years [12, 13]. Although CAR T-cell therapy can prolong survival, it also carries risks, including life-threatening adverse effects like cytokine release syndrome and immune effector cell-associated neurotoxicity syndrome, as well as long-lasting side effects such as persistent cytopenia and chronic susceptibility to infections [14, 15]. These physical complications can be accompanied by psychological and social burdens, including anxiety, depression, cognitive change [16], feelings of isolation [17], and financial strain [18]. Although HRQoL is increasingly gaining attention and is being evaluated as a secondary endpoint in several clinical trials and real-world CAR T-cell cohorts [19], most studies still solely focus on clinical outcomes such as response rates, progression-free survival, overall survival, and the severity of side effects [10, 11]. Meanwhile, research on HRQoL for recipients of CAR T-cell therapy is typically based on cross-sectional surveys or longitudinal studies that collect data at limited time points [19], falling short in capturing the temporal, continuous dynamics of HRQoL in relation to the dynamics of its interacting factors. To complement this, we have chosen the context of CAR T-cell therapy as the focus of our study.

A review shows that several mathematical and simulation models have been developed to study key clinical aspects of CAR T-cell therapy, such as tumor growth, the expansion and contraction of CAR T-cells, their interactions with cancer cells, and the risk of cytokine release syndrome [20]. However, our model is the first to link CAR T-cell therapy with HRQoL dynamics. We introduce a simulation model developed by integrating current research on CAR T-cell therapy with insights from subject matter experts. Our goal is to explore interactions among various factors that impact the HRQoL of cancer patients, assess its dynamics over time, and evaluate strategies aimed at enhancing HRQoL.

As a disclaimer, this model is designed to simulate potential outcomes rather than predict specific individual-level patient results; it is a tool for exploration, not a predictive clinical decision-making instrument. Additionally, while clinical practice often seeks specific answers on ‘how to’ improve HRQoL, the scope of this article is to examine overall dynamics and answer ‘what-if’ questions. Our model’s framework is deliberately designed to be generic, capturing the fundamental aspects of how a disease and its treatment influence well-being. The hope is that its versatile design enables the application to a wide range of treatments beyond CAR T-cell therapy, providing a new approach to expand research into the dynamic interactions that affect HRQoL.

## Methods

We developed an exploratory system dynamics simulation model, Q-PRIMA (health-related Quality of Life of Patients Receiving Immunotherapy: Modeling Analysis), to study HRQoL of cancer patients before and after CAR T-cell therapy. Our work is part of a consortium focused on the HRQoL of cancer patients receiving immunotherapy [21].

## Model development

### Model structure

We built the model based on our previously developed causal loop diagram that qualitatively represents factors affecting HRQoL of immunotherapy patients [1]. We established the model’s structure by building on the variables and interrelationships identified in the causal loop diagram. From this earlier qualitative work, which created a layered qualitative map capturing various factors—from family dynamics to drug development processes—we focused on short-term factors impacting the HRQoL of an individual receiving immunotherapy. To balance complexity and simplification, we considered variables with delays on comparable scales—decades for broader processes like drug development versus months for individual treatment decisions—allowing the quantified model to be more robust. We conducted a total of 17 additional interviews lasting between one and two hours, with three experts on CAR T-cell therapy and immunotherapy. Through the interviews, the model structure was modified iteratively to specify it for CAR T-cell therapy, which is one category within the broader field of immunotherapy.

The CAR T-cell treatment is complex and involves multiple steps, possible delays, and decision points. After a cancer (recurrence or progressive) diagnosis, a patient may be referred for CAR T-cell therapy if considered a candidate. Their eligibility is first assessed by a local team of physicians or a national tumor board, varying across countries. If approved, the patient’s T-cells are collected and genetically modified to create CAR T-cells. Following the preparation of these engineered cells, the patient’s suitability for the procedure is reassessed. If still deemed appropriate, the CAR T-cells are reinfused into the patient. Post-treatment, medical professionals evaluate the patients’ response to the therapy, categorizing outcomes as a complete response, partial response, or no response, which includes stable or progressive disease.

The patient’s well-being is integral to the CAR T-cell treatment, serving both as an input at various decision points—i.e., determining eligibility and guiding treatment supportive care adjustments—and as an output, influenced by whether they undergo the treatment and their subsequent response. This dual function positions the factors affecting a patient’s well-being as endogenous within the model, underscoring the necessity of interpreting it through feedback mechanisms. The model consists of feedback loops among variables that are either reinforcing or balancing. In a reinforcing loop, a change in one variable causes the other variables in the loop to compound the change in the same direction, leading to growth or decline. In contrast, in a balancing loop, a change in one variable leads to a change in the other variables in the loop in the opposite direction, leading to stability [22].

### Quantification

We developed model equations based on qualitative and quantitative information sourced from the literature, as well as insights gathered from the interviews. These equations are organized into two categories: (1) cancer and CAR T-cell treatment, and (2) psychological and physical well-being.

First, we utilized existing equations and studies on the dynamics of tumor growth, the expansion, contraction, and persistence of CAR T-cells, CAR T-cell interactions with cancer cells [23], and cytokine release syndrome resulting from this interaction [24]. Nonlinear mathematical models of CAR T-cell therapy have been applied in multiple studies [20] and across various CAR-T cell therapies for different indications [25].

Second, to address psychological well-being, we developed equations based on insights from interviews, focusing on stressors such as cancer treatment side effects and interactions among psychological adaptation, social support, and performance status of the patient. These equations were informed by established differential equation models of psychological well-being, integrating concepts such as the reservoir model of psychological regulation [26], emotional force, hedonic dampening (the adaptation to changes in state), and the well-being homeostatic system, which ensures equilibrium in well-being [27]. For physical well-being, we formulated equations reflecting principles of homeostasis and goal-seeking behavior [28].

In the model, we normalized parameters such as HRQoL, psychological well-being, physical well-being, tumor burden, side effect severity, and performance status to a scale ranging from zero to one. While it may not be suitable for clinical analysis and predictive purposes, this normalization facilitates consistent comparisons across different measures, simplifies the integration of these variables, and enhances the interpretability of the results. See online supplement for details and supporting references for these quantifications.

## Model testing

We followed best practices for assessing system dynamics models [22, 29]. First, we used experts’ opinions to build confidence in the model structure and behavior. Iterative interviews helped identify potential limitations and areas for improvement. To build confidence in the structure of the model, we iteratively modified the model until we achieved a consensus of expert opinions. Second, model projections were reviewed by experts to provide insights into model capability in representing clinical practice. Third, to test the robustness of formulations, we assessed the behavior of the model in extreme values.

### Sensitivity analysis

We assessed the sensitivity of the HRQoL in the model using univariate analysis of model parameters. This analysis involved altering each parameter by 10% below and above their original values. We then evaluated the impact of these variations by comparing the percent changes in HRQoL at the final simulation time against the outcomes from the model with the original parameters. More details are provided in the supplement.

## Model analysis

### Projected outcomes

The primary outcome of interest in our study is HRQoL. Initially, we analyzed the dynamics of HRQoL in conjunction with factors such as physical and psychological well-being, side effects, and tumor burden. Subsequently, we focused specifically on HRQoL, comparing its improvement across two intervention strategies for patients undergoing CAR T-cell therapy.

### Time horizon

Based on real-world studies of CAR T-cell therapy, time between indication for CAR T-cell therapy and tumor board approval has a median of 6 days [30] and the median time from the approval of treatment to infusion ranges from 46 to 68 days [30–33]. By calculating a weighted average from these studies, we have established a typical timeline of 60 days from the tumor board’s decision to the infusion. The start of the simulation represents the approval of CAR T-cell treatment. Time zero represents the infusion of CAR T-cells. Then, we run our projections over a time horizon, considering 180 days, a typical measurement interval used in clinical studies examining the HRQoL of patients receiving CAR T-cell therapy [19].

### Strategies

Strategies to improve HRQoL were identified from interviews and enhanced through the literature review. We analyze two specific strategies aimed at enhancing the HRQoL of patients receiving CAR T-cell therapy: reducing the time between approval and infusion and providing social support. Despite the expected benefits of early infusion, delays in this process still occur in practice. Similarly, given the clinical focus of treatment, social support, which could be of substantial additional value, is not always prioritized. Therefore, we chose to analyze these two strategies. Details of these strategies, including definitions, references, and examples, are outlined in Table 1.

We present HRQoL projections for two strategies across three distinct patient scenarios, defined based on HRQoL dynamics at the status quo without the improvement strategies. These scenarios include patients not initially eligible for infusion, those who experience a relapse, and patients with a complete response. To generate these scenarios, we adjusted the parameter representing the tumor growth rate, which reflects varying rates of disease progression—see details in the supplement.

**Table 1:** Strategies to improve HRQoL of patients receiving CAR T-cell therapy

	Description	Model adaptation	Real-life examples from cancer treatment and care
**Reducing delay in CAR T-cell infusion**	The delay represents the time between a patient’s indication for CAR-T cell treatment and the actual receipt of the infusion. This delay can arise as CAR-T cell therapy is typically a later-line treatment option for relapsed or refractory cancers, potentially leading to late referrals [34]. Once referred, the patient’s case is assessed by a tumor board, further extending the time before treatment can proceed. Following approval, the infusion process can still face a delay of 4–8 weeks due to the time required to produce CAR-T cells [35]. On the other hand, during this period, disease progression may render the patient ineligible for infusion [36]. This strategy aims to enhance the overall efficiency of the treatment timeline, thereby reducing wait times and potentially improving patient outcomes.	To demonstrate the impact of reducing delay, the time between the initiation of the simulation run and the infusion is reduced from its initial value of 60 days to 40 days. Examples of how this has been applied are in the right column.	**Improving insurance coverage**: Removing barriers and enhancing insurance coverage of immunotherapy will make immunotherapy more accessible to patients [37].
			**Educating the medical team on disease symptoms possible treatments**: Providing adequate medical education opportunities to healthcare providers to keep them up to date with new advancements will help in early referrals by these practitioners [38].
			**Reducing time to produce T cells**: Academic medical centers are attempting to make in-house CAR T-cell therapies with equal efficacy [39]. Spain has started decentralized CAR manufacturing, reducing the time to infuse T-cells [40].
**Increasing daily available social support**	We captured support by the total number of hours per day an individual gains for psychological adaptation by being relieved from other daily activities. We assumed that individuals require a certain time each day to manage tasks such as earning a living, cooking, cleaning, and coordinating social events. Additionally, individuals with a higher disease burden often experience reduced daily functioning and may require more time to complete these same tasks. This strategy focuses on increasing the availability of resources for those in need. When individuals utilize the available support, it can lighten their daily burdens, allowing patients more time to cope with and psychologically adjust to life changes, which in turn can enhance their overall well-being.	To capture social support enhancement, the available daily support variable increased from 2 h per day to up to 6 h per day. Examples of how social support availability can be provided for patients are in the right column.	**Connecting patients to community support groups**: There are cancer support groups that help cancer patients with a wide range of daily concerns, such as assistance with shopping [41], providing rides to treatment [42], covering childcare shifts [43], delivering nutritious meals to patients’ doorsteps [44], and arranging financial aid [45].
			**Providing health guidance when and where needed**: Professional support can be made more accessible for cancer patients through community physiotherapists, occupational therapists, dietitians, and nurses who assist with various needs such as exercise, daily activities, diet, and medical care [41]. In a study with telerehabilitation on functional impairment and pain the intervention group showed improved quality of life [46]. In another study intervention group with social care nurses showed higher psychological well-being than the control group [47].
			**Providing psychosocial support**: Psychosocial support should be considered in patients undergoing CAR T-cell therapy [48]. Reducing distress has been shown to lower symptoms burden and achieve higher levels of functioning [49]. Cancer patients within psychological intervention group demonstrated improved survival [50].

## Four measures of HRQoL dynamics

**Fig. 1:**
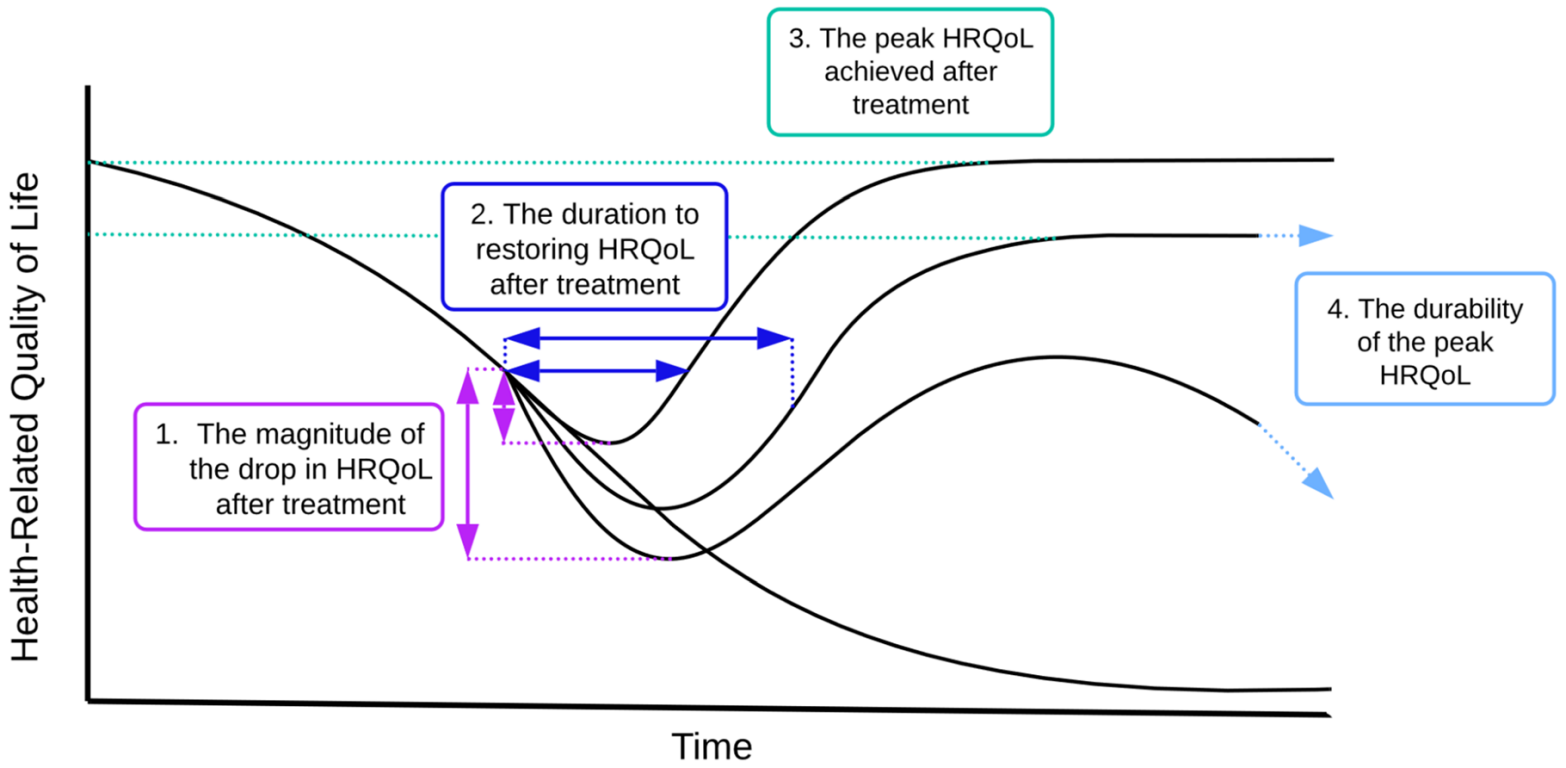
Four measures to analyze HRQoL dynamics over time. Note (1) the magnitude of the drop in HRQoL quantifies the extent of the decrease in HRQoL following infusion, specifically focusing on the minimum level observed; (2) the duration of restoring HRQoL, which examines how quickly HRQoL returns to its pre-infusion level after the initial decrease; (3) the peak level that HRQoL achieves after treatment; (4) the durability of the peak, which is a dichotomous measure that assesses whether the improvement in HRQoL maintains a steady, high-level equilibrium over time

To assess and compare the HRQoL projections for the patient groups discussed above at baseline and with the implementation of improvement strategies, we utilized four key measures, as illustrated in Fig. 1. We hope to position these four key measures in Fig. 1 as a framework for researchers analyzing HRQoL dynamics over time.

## Results

### Model overview

**Fig. 2:**
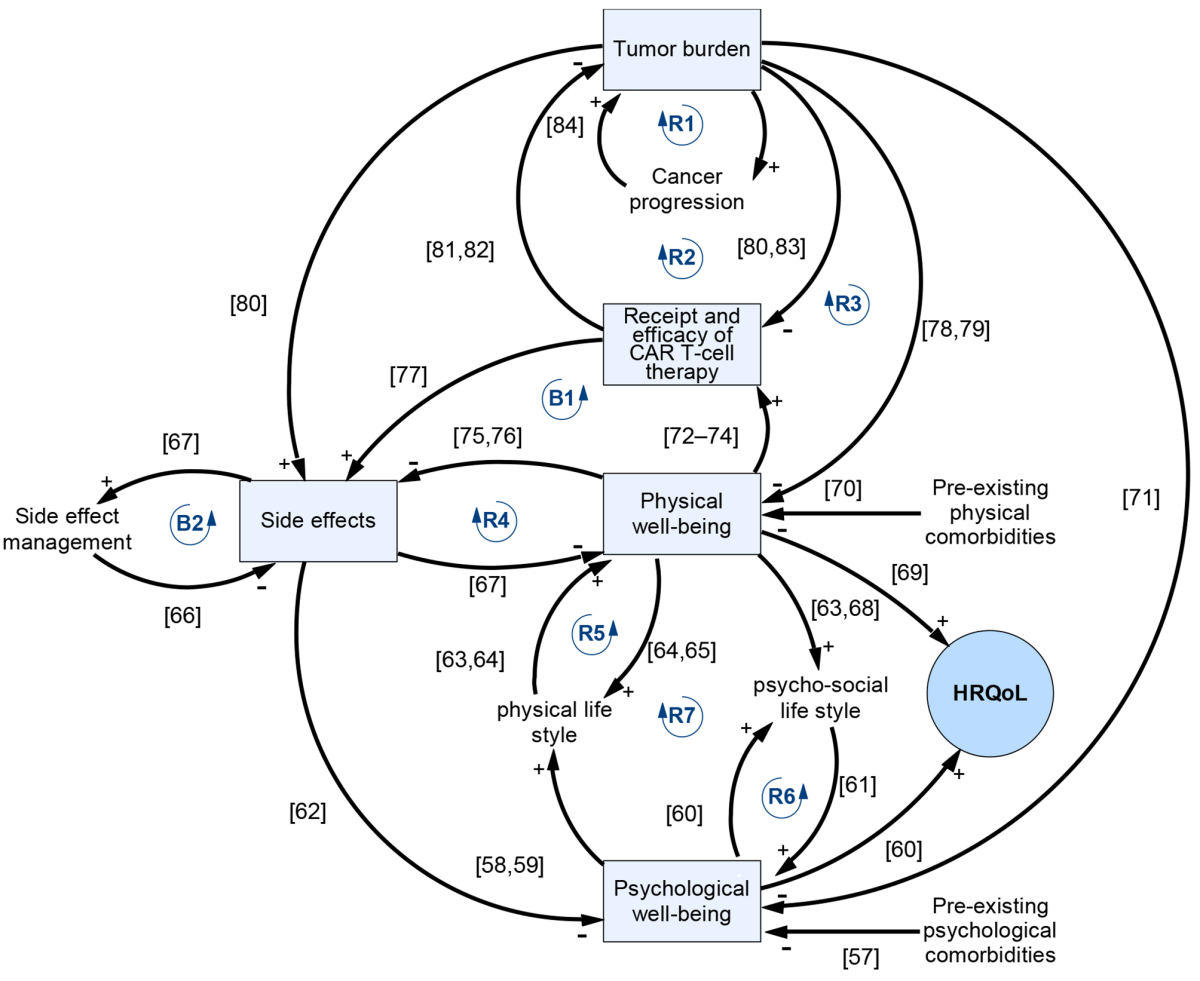
The high-level overview of the model, including the key variables, relationships, feedback loops and references. Note: The links connect two variables to each other, with the direction of the arrow indicating which variable influences the other. The ‘+’ and ‘-’ signs at the arrowhead denote whether an increase or decrease in one variable affects the variable it points to in the same or opposite direction, respectively. A collection of links that start and end with the same variable forms a loop. In a reinforcing loop (marked with ‘R’), one variable’s change impacts others in the loop until it circles back to amplify the initial change, driving growth or decline. In contrast, a balancing loop (marked with ‘B’) works to stabilize the system: one variable’s change affects others until it circles back to counter the initial change. The numbers accompanying ‘R’ and ‘B’ indicate the sequence in which they are discussed in the article

Figure 2 presents a high-level overview of the model, including the key variables, relationships, and feedback loops. The model includes five main elements represented as state variables (also known as stock variables, shown in boxes): physical well-being, psychological well-being, tumor burden, receipt and efficacy of CAR T-cell therapy, and side effects. The main outcome of interest, HRQoL, is represented by a circle.

The relationships between variables in the model are grounded in research, with citations represented in Fig. 2. The interconnections forming the feedback loops represent how these elements interact and influence each other dynamically over time, providing a systems view. In other words, by bringing these components together, the model offers a cohesive picture of the system’s overall behavior, allowing us to capture interactions that might otherwise be studied in isolation. This integrated approach enables a deeper understanding of the system’s dynamics and highlights potential leverage points for intervention.

The model consists of seven reinforcing feedback loops. First, a greater tumor burden indicates a more advanced stage of cancer, which in turn can lead to faster disease progression and increased tumor growth (loop-R1). Second, while patients are waiting for CAR T-cell treatment, they experience an increase in their tumor burden, which may decrease their likelihood of responding positively to the treatment (loop-R2). Third, as patients wait for treatment, increases in tumor burden worsen their physical well-being. This deterioration leads to a poorer performance status, which subsequently reduces their eligibility for infusion and exacerbates the tumor burden further (loop-R3). Fourth, patients who have a higher level of physical well-being usually experience milder side effects from the treatment, which in turn helps them maintain a higher physical well-being (loop-R4). Fifth, patients with higher levels of physical well-being engage in greater physical activity, which can improve their overall physical health (loop-R5). Sixth, patients with higher psychological well-being tend to be more socially active, which can enhance their psychological well-being (loop-R6). Finally, a higher level of physical well-being improves the ability to engage in higher psychosocial activity, resulting in improved psychological well-being; similarly, those with higher psychological well-being may also improve their physical well-being through increased physical activity (loop-R7). It is important to note that these loops can also operate in reverse; positive changes in one aspect of well-being can compound and enhance another, while negative changes can likewise lead to a reinforcing downward spiral.

The model also involves two balancing feedback loops, as presented in Fig. 2. CAR T-cell therapy is typically offered to cancer patients with better physical health, yet the side effects they encounter during treatment may cause a decline in physical well-being. Furthermore, the extent to which these side effects compromise physical well-being can impair survival outcomes and negatively impact the treatment’s efficacy (loop-B1). Second, as patients experience higher levels of side effects from CAR T-cell therapy, the medical team that consists of oncologists, nurses, and supportive care specialists identify and manage these adverse effects (loop-B2).

These feedback loops within our model demonstrate not just the individual effects but also how these loops collectively impact patient care and outcomes. The interactions among these loops build upon and influence each other, resulting in emergent dynamic behaviors that are further explored in the paper.

### Dynamics of HRQoL and inter-related factors

**Fig. 3:**
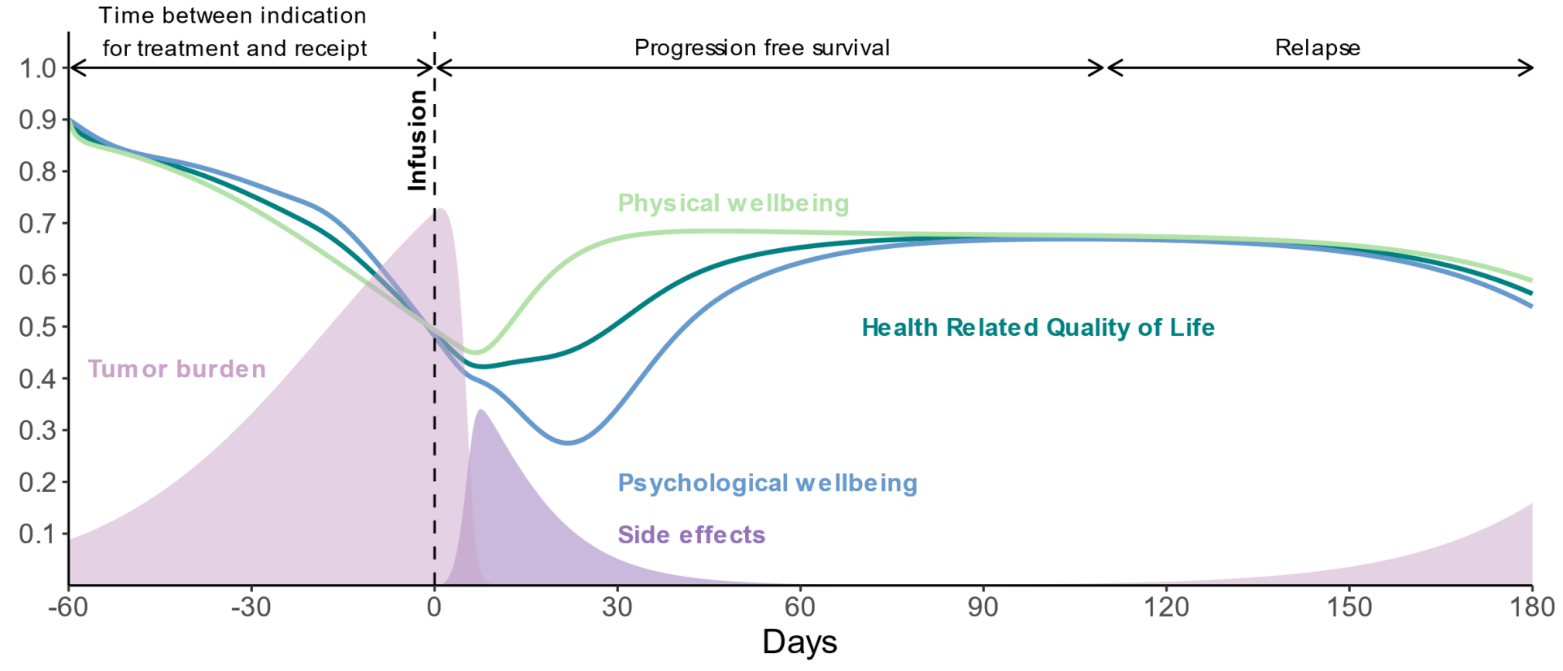
Example of dynamics of HRQoL factors for cancer patients receiving CAR T-cell treatment and timeline of key events. Note: As patients await CAR T-cell infusion, an increase in tumor burden might lead to a decline in physical well-being, which is followed by a deterioration in psychological well-being and HRQoL. Upon receiving the CAR T-cell infusion, patients might experience a swift reduction in tumor burden when they do respond. Despite this, the immediate period following the infusion can be characterized by a decline in HRQoL, largely driven by the onset of acute side effects. These side effects, which can be severe, initially worsen the patient’s overall condition. As healthcare providers manage these side effects, the impact is alleviated and leads to a recovery in physical well-being. Psychological well-being also begins to improve following this trend; however, this pace of recovery in psychological well-being can vary among individuals

Here we present an illustrative example to help readers understand the interaction of key factors shaping HRQoL dynamics, before we present the results of strategy analysis in the following section. For this example, we use a relapse scenario, as illustrated in Fig. 3. Building on the dynamics presented in this example, we will explore strategies for improving HRQoL in the following section.

### Strategy analysis

**Fig. 4:**
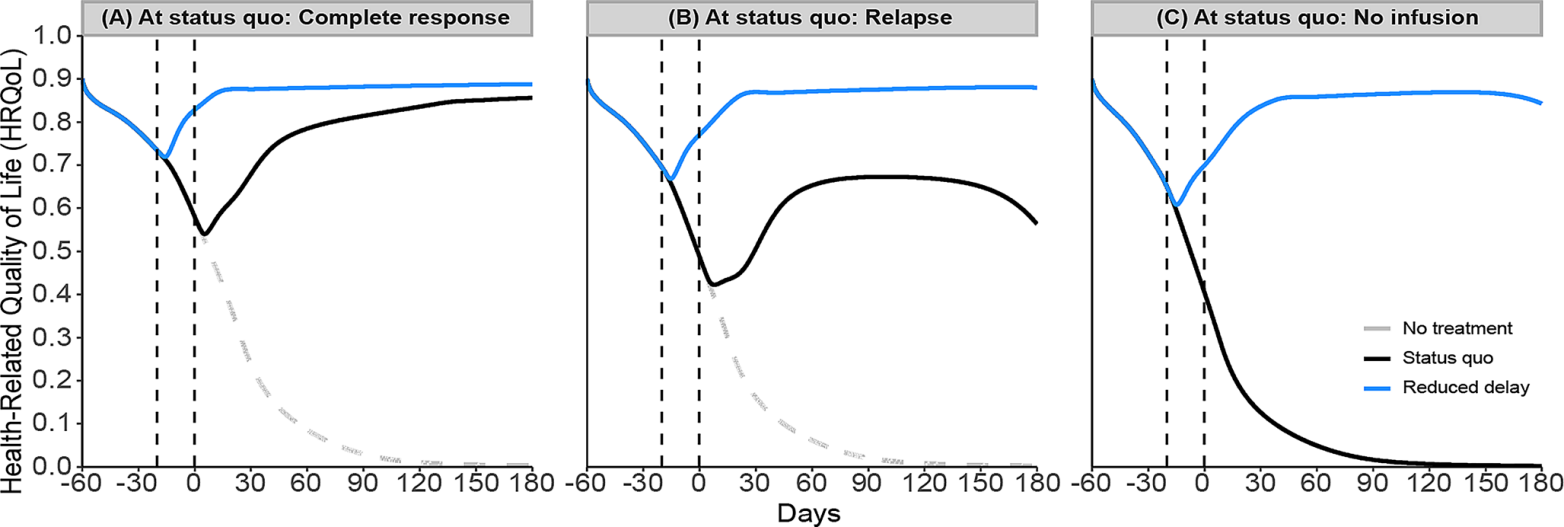
Effect of reducing delay in CAR T-cell infusion on HRQoL of patients under three scenarios. Initially, we compare the status quo dynamics for all scenarios (black lines; Fig. 4). In the complete response scenario (panel-**A**), HRQoL decreases post-infusion but quickly recovers to pre-infusion levels and eventually surpasses them, maintaining a high and durable level thereafter. However, in the relapse scenario (panel-**B**), the decrease in HRQoL is more pronounced, and recovery to pre-infusion levels takes longer. Although HRQoL eventually exceeds the initial level post-infusion, this heightened level is not durable over time. In contrast, the no-infusion scenario (panel-**C**) shows a progressive deterioration in HRQoL, ultimately leading to death

Figure 4 presents the dynamics of HRQoL for three distinct patient scenarios—complete response, relapse, and no infusion—each based on their HRQoL dynamics at the status quo, which is the current or usual state of health and treatment management for each particular individual.

To restate, the HRQoL dynamics of all scenarios are driven by the feedback loops presented in Fig. 2 and the dynamics described in Fig. 3, and we do not make separate assumptions for scenarios. Each scenario—whether involving patients not initially eligible for infusion, those experiencing a relapse, or those achieving a complete response—is modeled using the same parameters, with only the tumor growth rate adjusted to reflect different rates of disease progression. In other words, for scenarios like relapse and complete response, the feedback loops suggest that higher tumor progression leads to greater tumor burden, which in turn causes a quicker decline in performance status. This sequence is linked to lower treatment efficacy, potentially leading to a relapse. Despite the relapse scenario having the same side effect management and physical and psychosocial lifestyle parameters as in the complete response scenario, the bigger drop in HRQoL post-treatment may not be fully mitigated by the existing support systems, resulting in delayed recovery. Which in turn adversely affects physical and psychosocial lifestyles resulting in lower HRQoL than might otherwise be expected.

When comparing each patient scenario at the status quo against the reduced delay strategy (blue lines; Fig. 4), varying improvements are observed. For patients who experienced a complete response (panel-**A**), reducing the delay decreases the magnitude of the initial drop in HRQoL, accelerates the recovery to pre-infusion levels, and achieves a higher level of HRQoL with no change to its durability. Similarly, in the relapse scenario (panel-**B**), reducing the delay lessens the initial drop in HRQoL and shortens the recovery time, enhancing the peak HRQoL achieved after treatment. Additionally, it contributes to the durability of this improvement. In the no-infusion case (panel-**C**), while patients maintain the status quo without receiving an infusion, the reduced delay strategy could potentially allow these patients to qualify for infusion, resulting in an increase in HRQoL post-infusion, provided they respond to the therapy.

**Fig. 5:**
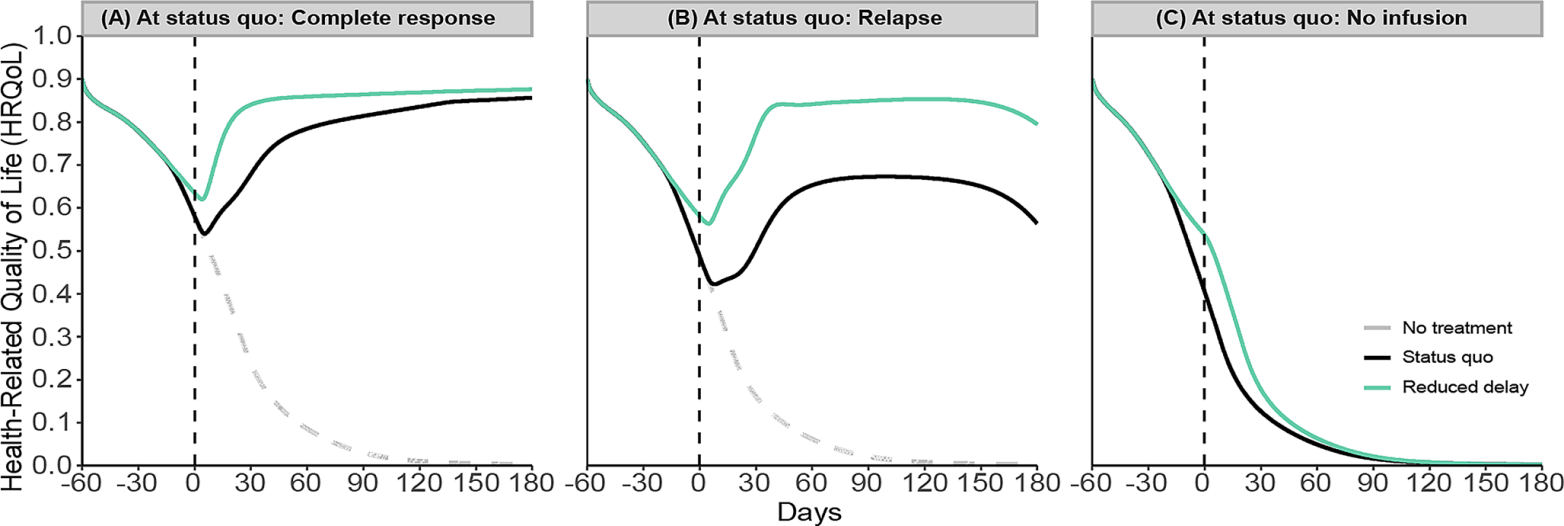
Effect of social support on HRQoL of patients receiving CAR T-cell therapy under three patient scenarios

Next, we compare each patient scenario at the status quo against the social support strategy (green lines; Fig. 5). In the scenario where patients achieve a complete response (panel-**A**), the introduction of social support may mitigate the severity of HRQoL declines post-treatment, quicken the recovery to pre-infusion HRQoL levels, and elevate HRQoL to a higher, durable level. In the relapse scenario (panel-**B**), social support reduces the initial HRQoL drop, shortens the recovery period, and enhances the level of HRQoL achieved after treatment. Despite these improvements, there is no enhancement in the long-term durability of HRQoL for relapsed patients. Meanwhile, in the no-infusion scenario (panel-**C**), although social support slightly improves HRQoL, this strategy alone is insufficient to alter the overall trajectory or durability of HRQoL.

## Discussion

Understanding the complexities and dynamics of HRQoL is important in order to improve it, and simulation modeling provides a powerful tool for this exploration. In this study, we have made three contributions. Firstly, we developed a simulation model to analyze HRQoL dynamics through a systems approach. Second, we demonstrated how HRQoL dynamics can be compared and analyzed over time in conjunction with other interacting factors. Thirdly, we specifically applied this model to CAR T-cell therapy, exploring the potential impacts of various HRQoL improvement strategies: reducing the delay in infusion and enhancing social support.

Our model, intentionally designed for broad applicability, examines key HRQoL interactions, including physical and psychological well-being, disease burden, treatment receipt and efficacy, side effects, and their management. We introduced metrics to dynamically evaluate and compare HRQoL: post-treatment HRQoL decline level, recovery time to pre-treatment HRQoL level, post-treatment HRQoL peak, and the durability of the peak.

In the context of CAR T-cell therapy, our study explored how increases in tumor burden while patients await treatment can lead to a deterioration in physical well-being, subsequently affecting psychological well-being and HRQoL. Despite a reduction in tumor burden after infusion, HRQoL may continue to decline due to side effects that initially worsen the patient’s condition. As these side effects are managed, improvements in both physical and psychological well-being are observed. The well-being of patients plays a critical role in the treatment process, influencing eligibility decisions and necessary treatment-supportive care adjustments. By treating well-being as an endogenous variable, our model captured feedback, e.g., how increased tumor burden due to delays in treatment can exacerbate side effects or reduce treatment efficacy, potentially leading to relapse or low well-being even rendering patients ineligible for receiving infusion in the first place. We varied disease progression to examine three distinct patient scenarios: those not initially eligible for treatment, patients experiencing a relapse, and patients achieving a complete response. Subsequently, we evaluated the impact of two strategies aimed at improving HRQoL—reducing the delay to infusion and enhancing social support—across these scenarios.

Scenario analysis demonstrated that reducing the delay in CAR T-cell infusion can be an effective strategy to improve HRQoL. Such delay reduction can be achieved through various strategies, e.g., removing financial barriers to CAR T-cell access [18], awareness of CAR T-cell therapy as a treatment option for patients, timely referral and speeding up the T-cell manufacturing process [18]. For patients who experience a complete response, reducing the delay minimizes the initial drop in HRQoL, speeds up the recovery to pre-infusion levels, and results in a higher, durable HRQoL. In the relapse scenario, this approach also lessens the initial HRQoL drop and shortens recovery time, while enhancing the peak HRQoL achieved after treatment and contributing to its durability. Even in scenarios where patients do not initially qualify for an infusion, reducing delays could potentially make them eligible, thereby improving their post-infusion HRQoL.

Our findings are consistent with previous research, which has shown that delays in treatment initiation can impact patient outcomes [36], with a delay of just one month leading to increased mortality rates [51]. When treatment is initiated earlier with a relatively lower tumor burden, patients are more likely to experience fewer side effects [52] and higher treatment efficacy [36, 52]. Moreover, a shorter delay in treatment initiation can improve patients’ overall HRQoL by maintaining their physical well-being and eligibility for CAR T-cell therapy [53], which can ultimately result in significant improvements in survival [53, 54]. Our study extends this understanding by demonstrating how the interacting factors of HRQoL change dynamically over time and how they influence each other collectively. It also provides a way to compare patient cases, including those who experienced a complete response, relapse, or no response. In real-world scenarios, it is infeasible or unethical to know what would have happened to a patient if they had received an intervention at a different time. However, simulation models allow us to explore these “what-if” scenarios, offering insights into the potential outcomes of various HRQoL improvement strategies.

By comparing each patient scenario against the status quo with the social support strategy, we demonstrated that in cases where patients achieve a complete response, social support mitigates the severity of HRQoL declines post-treatment, accelerates recovery to pre-infusion HRQoL levels, and elevates HRQoL to a higher, durable level. In the relapse scenario, social support similarly reduces the initial HRQoL drop, shortens the recovery period, and enhances the level of HRQoL achieved after treatment. This aligns with previous observational studies that have shown that strengthening the psychosocial well-being of patients improves their overall quality of life [55]. Although our strategy analysis indicated that social support did not enhance the long-term durability of HRQoL for the relapsed scenario and did not alter the trajectory or durability of HRQoL in the no-infusion scenario, there are studies demonstrating that psychological interventions can improve survival rates for cancer patients [50]. Our model could account for these outcomes through interactions and feedback structures. For example, increased social support can lead to improved psychological well-being and promote healthier lifestyles and better physical health. This improvement in physical health can enhance performance status, thereby increasing the likelihood of patients receiving and responding to more effective treatment. Therefore, our model should be viewed not as a predictive tool for specific outcomes but as a tool for exploring the potential impacts of various interventions on patient well-being. Such explorations can include alternative scenario analysis, such as observing the effect of offering support at different stages—before, during, or after treatment—to assess how the timing of these interventions affects different components of well-being as well as looking at the combined effect of strategies.

Our analysis has limitations. First, due to the lack of data from real-world patients, our model is based on estimates from literature and limited subject matter interviews, as well as assumptions that may not fully reflect the diverse patient populations and treatment contexts. For instance, we use parameters for B-cell acute lymphoblastic leukemia patients, but critical factors like tumor killing and growth rates, and initial tumor burden can vary across different cancer types, requiring caution when generalizing findings. This reliance on secondary data may result in a less precise representation of real-world dynamics. Second, we were unable to attain disaggregated clinical data on a continuous time scale, and the use of summarized data potentially overlooking short-term fluctuations in patient outcomes may have led to a loss of precision and detail in our analysis. Third, we did not consider the hyper-progression dynamic after CAR T-cell infusion due to limited information. Fourth, we did not include patient characteristics such as weight, age, and sex and their number of prior therapies, genetic markers, histology, and scans; however, in real life the outcomes could vary depending on these factors. Therefore, caution should be taken when interpreting our findings for different patient populations and healthcare systems. Despite these limitations, this study establishes a foundation for future HRQoL research and modeling. Future research could focus on integrating real-world longitudinal patient-specific data into the simulation model to improve prediction accuracy. Additionally, using variable variations to represent patient cohorts could provide opportunities for more detailed analyses, such as cost-benefit analysis.

In conclusion, we offer a novel systems-based approach to understanding the dynamics of HRQoL. Through simulation modeling, we can explore the effects of different strategies on HRQoL, while also capturing the dynamic interactions between its key components. This approach provides a powerful tool for investigating aspects of HRQoL that are difficult to measure in real-world settings. Our model is adaptable to other diseases beyond CAR T-cell therapy, and the four metrics introduced here can be applied in future studies to better assess and understand HRQoL dynamics.

## Electronic supplementary material

Below is the link to the electronic supplementary material.

Supplementary Material 1

## Data Availability

Data and analysis details are reported in the online supplementary document.
