# Supplementary material for: Health-related quality of life dynamics: modeling insights from immunotherapy
Source: Qual Life Res. 2024 Oct 30;34(1):273–86. doi: 10.1007/s11136-024-03810-0 (PMC11802606; doi:10.1007/s11136-024-03810-0)
Supplement: Supplementary file 1 — Supplementary Material 1 [file 11136_2024_3810_MOESM1_ESM.docx]

*Supplementary file for*

**Health-related** **Quality of Life Dynamics: Modeling Insights from Immunotherapy**

Contents

[Model Overview 2](#_Toc171082608)

[Model details: files, equations, and parameters 5](#_Toc171082609)

[Model Testing 6](#_Toc171082610)

[Sensitivity Analysis 7](#_Toc171082611)

In this supplementary document, we present a detailed description as well as resources of our simulation model Q-PRIMA (health-related Quality of Life of Patients Receiving Immunotherapy: Modeling Analysis). Specifically, we provide information about model parameters, simulation specifications, and additional analyses. We also report additional details on model testing, including extreme condition tests and sensitivity analyses, which were conducted to build confidence in our model.

## Model overview

**Table S1** presents the description and references of the relationships in the model. For convenience, we copied the model illustration from the article below in **Figure S1**.

[14]

[3,4]

[1,5]

[1]

[6,7]

[2]

[11,12]

[8–10]

[13]

[25]

[28]

[22]

[17,18]

[27]

[16,21]

[13]

[16,17]

[23]

[24]

[19,20]

[23]

[15]

[26]

**Figure S1:** The high-level overview of the model, including the key variables, relationships, feedback loops, and references. Loops labeled as R# and B# are discussed in the article.

**Table S1:** Details on the relationships coming from references

| Causal relationship | Description | Sources |
| --- | --- | --- |
| Increase in tumor burden increases side effects | Greater baseline tumor volume is associated with poorer CAR T-cell survival outcomes. High tumor burden has been associated with higher treatment-associated side effects e.g., high-grade cytokine release syndrome (CRS) toxicities. | [1] |
| Increase in receipt and efficacy of CAR T-Cell therapy increases side effects | CAR T-cell clinical trials report severe, life-threatening events in patients. | [2] |
| Increase in receipt and efficacy of CAR T-Cell therapy decreases tumor burden | The authors reviewed several clinical trials and summarized the percent overall response after CAR T-cell therapy. This is a measure of a decrease in tumor burden. | [3] |
|  | Increasing the dose of CAR T-cell therapy increases objective response rates up to a certain threshold. | [4] |
| Increase tumor burden decreases in receipt and efficacy of CAR T-Cell therapy | 27 patients out of 404 enrolled could not get CAR T-cell therapy after enrollment due to either progressive disease or death due to cancer. | [5] |
|  | DLBCL patients with lower tumor burden post bridging therapy had improved progression-free survival indicating greater baseline tumor volume is associated with poorer CAR T outcomes. | [1] |
| Increased tumor burden decreases physical well-being | Tumor burden causes a decrease in physical functioning by causing cachexia, a loss of skeletal muscle mass disease. | [6] |
|  | Cancer patients suffering from primary non-metastatic, metastatic, and recurrent tumors have reported lower physical function over time. | [7] |
| Increased physical well-being increases receipt and efficacy of CAR T-Cell therapy | In most clinical trials, patients with better performance status receive chemotherapy receipts (ECOG 0-1) compared to patients with poor performance status. | [8] |
|  | ECOG score ≥2 is a risk factor for less than 3-year overall survival compared to ECOG 0-1. | [9] |
|  | CAR T-cell recipient patients with ECOG 0-2 had an overall response rate of 79.3% compared to patients with ECOG 3-4 (75.0%). | [10] |
| Increased physical well-being decreases side effects | Good performance status predicts safe outcomes. | [11] |
|  | Exercise has a powerful effect on cancer-related fatigue due to therapy; fatigue levels were 40% to 50% lower in exercising participants. | [12] |
| Increase in side effects decreases physical well-being | CAR T-cell-related toxicities include CRS, neurotoxicity syndrome, B-cell aplasia, cytopenia, and infections decreasing physical well-being. | [13] |
| Increase in tumor burden increases tumor burden | Larger tumor burden was associated with more aggressive tumor growth. | [14] |
| Increase side effect decreases psychological well-being | Immunotherapies have the most common and clinically relevant psychological side effects. | [15] |
| Increase in physical lifestyle increases physical well-being | Physical activity and exercise positively affect physical function. | [16,17] |
| Increase in physical well-being increases physical lifestyle | Increased physical activity is related to better physical functioning and reduced fatigue and bodily pain. | [17] |
|  | High body mass index is related to an increased desire to avoid exercise and potentially decrease physical activity levels. | [18] |
| Increase in psychological well-being increases in physical lifestyle | Posttraumatic stress disorder and depression negatively impact physical health. | [19] |
|  | Depression increases sedentary lifestyle risk and is related to a decreased level of physical exercise. | [20] |
| An increase in physical well-being increases psycho-social lifestyle | Moderate-to-vigorous physical activity increases physical well-being and decreases distress from baseline. | [21] |
|  | Physical activity and exercise positively affect emotional well-being. | [16] |
| Increase in physical well-being increases HRQoL | Systemic review shows that physical activity improves QOL. | [22] |
| Increase in psychological well-being increases HRQoL | Interventions affecting the psychosocial well-being of the patient, improve their psychological well-being and QOL. | [23] |
| Increase in psychological well-being increases psycho-social lifestyle | Interventions enhancing psychosocial health of African American female breast cancer survivors enhance their psychological well-being and QOL. | [23] |
| Increase in psychosocial lifestyle increases psychological well-being | Providing consistent mental-health training, organizational support, and emotional and psychological support, including peer-support and counseling, therapy prevent and reduce mental health issues. | [24] |
| Pre-existing physical comorbidities decreases physical well-being | Patients hospitalized with pneumonia, stroke, and other illness that required prolonged mechanical ventilation are highly susceptible to recurrent infections, sepsis, and death | [25] |
| Increase in pre-existing psychological comorbidities decrease psychological well-being | Depression and anxiety are highly comorbid, decreasing phycological well-being. | [26] |
| Increase in side effect increases side effect management | Patients with moderate to severe CAR T-cell associated neurotoxicity syndrome are managed with corticosteroids and supportive care. | [13] |
| Increase in side effect management decreases side effects | Early administration and/or short corticosteroid cycles are associated with the resolution of neurological toxicities. | [27] |
| Increase in tumor burden decreases psychological well-being | Clinical depression is increasing in cancer patients. | [28] |

## Model details: files, equations, and parameters

Model files were created using Vensim software. All Vensim files to run the model are available on GitHub at: <https://github.com/zhasgul/Q-PRIMA.git>

Vensim software is available through Ventana Systems, Inc.: <https://www.ventanasystems.com>

Model equations, parameters, their descriptions, and references are reported in an Excel file, available at: <https://github.com/zhasgul/Q-PRIMA.git>

Supplementary text for model parameters

Physical well-being is scaled between zero and one, where zero represents death, and one indicates perfect health. Similarly, for psychological well-being, one denotes the best well-being, and zero represents the worst possible condition. We calculated HRQoL as the average of these psychological and physical well-being scores, which is similar to other survey-based analyses that use summarized information such as Short Form Health Survey (SF-12) [29] and PROMIS global physical and mental health scale [30]. Tumor burden is scaled such that zero indicates no tumor presence, while one represents the most severe disease. The scale often used in cancer literature for side effect severity is grades 1-2 for mild effects and grades 3-4 for severe effects. In our model, a side effect severity of zero corresponds to no side effects, and one corresponds to grade 4 severity. Additionally, a grade 5, indicating death, is modeled as physical well-being dropping to zero. Performance status, often assessed by the Eastern Cooperative Oncology Group (ECOG) scale, measures disease progression’s impact on daily activities, where zero represents full activity, four indicates complete disability, and five represents death [31]. In our model, performance status is converted to a value between zero and one, with one representing the highest performance and zero the lowest. Patients with an ECOG score of two or lower are generally considered fit for CAR T-cell therapy [32]. Hence, we assumed that patients with performance status higher than the 0.6 threshold are considered eligible for the CAR T-cell infusion.

To inform our model inputs such as tumor growth rate, CAR T-cell expansion and apoptosis rates, and cytokine production rate, we collected quantitative data from previously validated modeling studies on CAR T-cell therapies; these studies were calibrated against clinical trial data for B-cell acute lymphoblastic leukemia (B-ALL) [33–35]. We also gathered additional input from the literature on clinical trials and real-world studies. We calculated an average initial performance status from clinical studies, which resulted in a value of 0.9 [36–38]. We assumed that aside from the tumor growth the model starts at an equilibrium—in the absence of the tumor the physical and psychological wellbeing remains at homeostasis at their initial values. Hence, we initiated physical well-being and psychological well-being equal to initial performance status of 0.9. For initial tumor burden, we used 8.75%, which was the median initial bone marrow blasts for B-ALL patients receiving CAR T-cell therapy in a cohort of 110 patients [39]. Due to uncertainty in these assumptions, we will vary them in our sensitivity analyses.

## Model testing

Here, we detail our model testing protocols under extreme conditions:

- If tumor burden reaches its highest value of one, the patient’s physical well-being shoul drop to zero and stays there.
- Upon patient death (physical well-being at zero), their HRQoL should also fall to zero. On the other hand, if psycholgical wellbeing drops to zero, HRQoL does not need to drop zero immediately.
- A tumor burden that starts at zero should not increase.
- Patients with no tumor burden should not undergo immunotherapy.
- Patients below the performance status threshold should not receive CAR T-cell therapy.
- Without a positive infusion decision, patients will not receive immunotherapy, and their tumor burden should not decrease.
- Patients not receiving immunotherapy should experience no related side effects and side effect management.
- Severe side effects causing death should reduce physical well-being to zero permanently.
- CAR T-cell therapy should be given only once; patients who relapse should not receive further treatment.

## Sensitivity analysis

Results of sensitivity are presented in **Figure S2**.


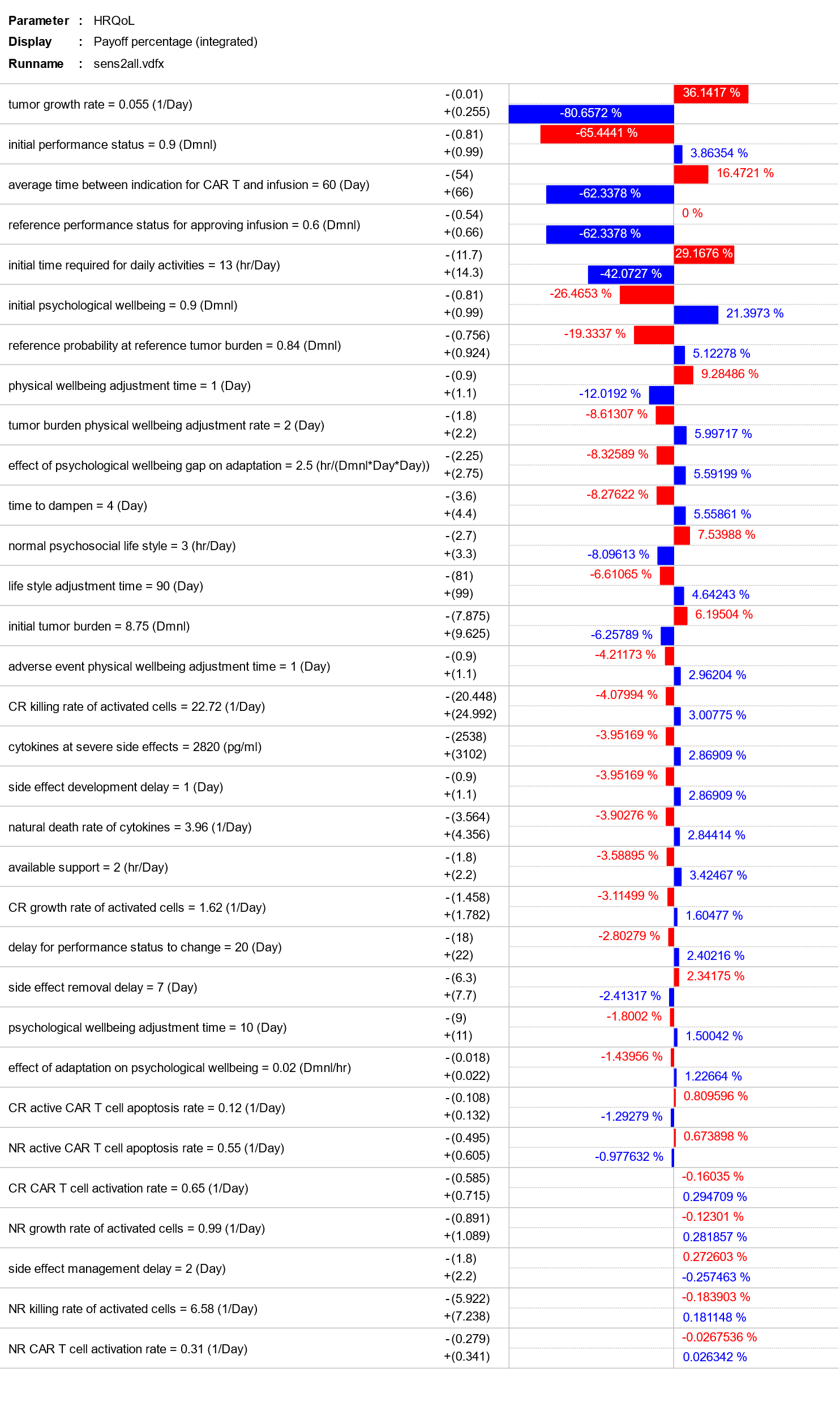


**Figure S2:** Tornado plot for univariate sensitivity analysis of model parameters

**References:**

1 Hubbeling H, Silverman EA, Michaud L, *et al.* Bridging Radiation Rapidly and Effectively Cytoreduces High-Risk Relapsed/Refractory Aggressive B Cell Lymphomas Prior to Chimeric Antigen Receptor T Cell Therapy. *Transplant Cell Ther*. 2023;29:259.e1-259.e10. doi: 10.1016/J.JTCT.2022.12.021

2 Sterner RC, Sterner RM. CAR-T cell therapy: current limitations and potential strategies. *Blood Cancer J*. 2021;11. doi: 10.1038/S41408-021-00459-7

3 Sermer D, Brentjens R. CAR T-cell therapy: Full speed ahead. *Hematol Oncol*. 2019;37 Suppl 1:95–100. doi: 10.1002/HON.2591

4 Rotte A, Frigault MJ, Ansari A, *et al.* Dose-response correlation for CAR-T cells: a systematic review of clinical studies. *J Immunother Cancer*. 2022;10. doi: 10.1136/JITC-2022-005678

5 Kuhnl A, Roddie C, Kirkwood AA, *et al.* A national service for delivering CD19 CAR-Tin large B-cell lymphoma - The UK real-world experience. *Br J Haematol*. 2022;198:492–502. doi: 10.1111/BJH.18209

6 Bland KA, Harrison M, Zopf EM, *et al.* Quality of Life and Symptom Burden Improve in Patients Attending a Multidisciplinary Clinical Service for Cancer Cachexia: A Retrospective Observational Review. *J Pain Symptom Manage*. 2021;62:e164–76. doi: 10.1016/J.JPAINSYMMAN.2021.02.034

7 Siddiqi A, Given CW, Given B, *et al.* Quality of life among patients with primary, metastatic and recurrent cancer. *Eur J Cancer Care (Engl)*. 2009;18:84–96. doi: 10.1111/J.1365-2354.2008.01021.X

8 Shah BD, Ghobadi A, Oluwole OO, *et al.* KTE-X19 for relapsed or refractory adult B-cell acute lymphoblastic leukaemia: phase 2 results of the single-arm, open-label, multicentre ZUMA-3 study. *Lancet*. 2021;398:491–502. doi: 10.1016/S0140-6736(21)01222-8

9 Puckrin R, Stewart DA, Shafey M. Real-World Eligibility for Second-Line Chimeric Antigen Receptor T Cell Therapy in Large B Cell Lymphoma: A Population-Based Analysis. *Transplant Cell Ther*. 2022;28:218.e1-218.e4. doi: 10.1016/j.jtct.2022.01.024

10 Du J, Wei R, Jiang S, *et al.* CAR-T cell therapy targeting B cell maturation antigen is effective for relapsed/refractory multiple myeloma, including cases with poor performance status. *Am J Hematol*. 2022;97:933–41. doi: 10.1002/AJH.26583

11 Frey N, Porter D. Cytokine Release Syndrome with Chimeric Antigen Receptor T Cell Therapy. *Biol Blood Marrow Transplant*. 2019;25:e123–7. doi: 10.1016/J.BBMT.2018.12.756

12 Mock V, Abernethy AP, Atkinson A, *et al.* Cancer-related fatigue clinical practice guidelines in oncology. *JNCCN Journal of the National Comprehensive Cancer Network*. 2007;5:1054–78. doi: 10.6004/JNCCN.2007.0088

13 Santomasso BD, Nastoupil LJ, Adkins S, *et al.* Management of Immune-Related Adverse Events in Patients Treated With Chimeric Antigen Receptor T-Cell Therapy: ASCO Guideline. *J Clin Oncol*. 2021;39:3978–92. doi: 10.1200/JCO.21.01992

14 Wu C Te, Huang YC, Chen WC, *et al.* Effect of Tumor Burden on Tumor Aggressiveness and Immune Modulation in Prostate Cancer: Association with IL-6 Signaling. *Cancers (Basel)*. 2019;11. doi: 10.3390/CANCERS11070992

15 Péter K, Gitta P, Krisztina M, *et al.* Psychological aspects of immunotherapies in the treatment of malignant melanoma. *Magy Onkol*. 2016;60:22–7.

16 Wright KD, Pepper GA, Caserta M, *et al.* Factors that influence physical function and emotional well-being among Medicare-Medicaid enrollees. *Geriatr Nurs*. 2015;36:S16. doi: 10.1016/J.GERINURSE.2015.02.022

17 Alfano CM, Smith AW, Irwin ML, *et al.* Physical activity, long-term symptoms, and physical health-related quality of life among breast cancer survivors: A prospective analysis. *J Cancer Surviv*. 2007;1:116. doi: 10.1007/S11764-007-0014-1

18 Vartanian LR, Shaprow JG. Effects of weight stigma on exercise motivation and behavior: a preliminary investigation among college-aged females. *J Health Psychol*. 2008;13:131–8. doi: 10.1177/1359105307084318

19 Malone C, Wachholtz A. The Relationship of Anxiety and Depression to Subjective Well-Being in a Mainland Chinese Sample. *J Relig Health*. 2018;57:266. doi: 10.1007/S10943-017-0447-4

20 Roshanaei-Moghaddam B, Katon WJ, Russo J. The longitudinal effects of depression on physical activity. *Gen Hosp Psychiatry*. 2009;31:306–15. doi: 10.1016/J.GENHOSPPSYCH.2009.04.002

21 Awick EA, Ehlers DK, Aguiñaga S, *et al.* Effects of a randomized exercise trial on physical activity, psychological distress and quality of life in older adults. *Gen Hosp Psychiatry*. 2017;49:44–50. doi: 10.1016/J.GENHOSPPSYCH.2017.06.005

22 Marquez DX, Aguinãga S, Vásquez PM, *et al.* A systematic review of physical activity and quality of life and well-being. *Transl Behav Med*. 2020;10:1098. doi: 10.1093/TBM/IBZ198

23 Gordon T, Lee LJ, Tchangalova N, *et al.* Psychosocial protective interventions associated with a better quality of life and psychological wellbeing for African American/Black female breast cancer survivors: an integrative review. *Support Care Cancer*. 2022;30:1093–114. doi: 10.1007/S00520-021-06425-0

24 Zaçe D, Hoxhaj I, Orfino A, *et al.* Interventions to address mental health issues in healthcare workers during infectious disease outbreaks: A systematic review. *J Psychiatr Res*. 2021;136:319–33. doi: 10.1016/J.JPSYCHIRES.2021.02.019

25 Baldwin MR. Measuring and Predicting Long-Term Outcomes in Older Survivors of Critical Illness. *Minerva Anestesiol*. 2015;81:650.

26 Kessler RC, Berglund P, Demler O, *et al.* The epidemiology of major depressive disorder: results from the National Comorbidity Survey Replication (NCS-R). *JAMA*. 2003;289:3095–105. doi: 10.1001/JAMA.289.23.3095

27 Rice J, Nagel S, Randall J, *et al.* Chimeric Antigen Receptor T Cell-Related Neurotoxicity: Mechanisms, Clinical Presentation, and Approach to Treatment. *Curr Treat Options Neurol*. 2019;21. doi: 10.1007/S11940-019-0580-3

28 Schumacher JR, Palta M, Loconte NK, *et al.* Characterizing the psychological distress response before and after a cancer diagnosis. *J Behav Med*. 2013;36:591–600. doi: 10.1007/S10865-012-9453-X

29 Ware JE, Kosinski M, Keller SD. A 12-Item Short-Form Health Survey: construction of scales and preliminary tests of reliability and validity. *Med Care*. 1996;34:220–33. doi: 10.1097/00005650-199603000-00003

30 Hays RD, Schalet BD, Spritzer KL, *et al.* Two-item promis® global physical and mental health scales. *J Patient Rep Outcomes*. 2017;1:1–5. doi: 10.1186/S41687-017-0003-8/TABLES/2

31 Zubrod CG, Schneiderman M, Frei E, *et al.* Appraisal of methods for the study of chemotherapy of cancer in man: Comparative therapeutic trial of nitrogen mustard and triethylene thiophosphoramide. *J Chronic Dis*. 1960;11:7–33. doi: 10.1016/0021-9681(60)90137-5

32 Amini L, Silbert SK, Maude SL, *et al.* Preparing for CAR T cell therapy: patient selection, bridging therapies and lymphodepletion. *Nat Rev Clin Oncol*. 2022;19:342–55. doi: 10.1038/S41571-022-00607-3

33 Liu L, Ma C, Zhang Z, *et al.* Computational model of CAR T-cell immunotherapy dissects and predicts leukemia patient responses at remission, resistance, and relapse. *J Immunother Cancer*. 2022;10:5360. doi: 10.1136/jitc-2022-005360

34 Hardiansyah D, Ng CM. Quantitative Systems Pharmacology Model of Chimeric Antigen Receptor T-Cell Therapy. *Clin Transl Sci*. 2019;12:343–9. doi: 10.1111/CTS.12636

35 Zhang Z, Liu L, Ma C, *et al.* A Computational Model of Cytokine Release Syndrome during CAR T-cell Therapy. *Adv Ther (Weinh)*. 2022;5. doi: 10.1002/ADTP.202200130

36 Kamal M, Joseph J, Greenbaum U, *et al.* Patient-Reported Outcomes for Cancer Patients with Hematological Malignancies Undergoing Chimeric Antigen Receptor T Cell Therapy: A Systematic Review. *Transplant Cell Ther*. 2021;27:390.e1-390.e7. doi: 10.1016/J.JTCT.2021.01.003

37 Wang XS, Srour SA, Mendoza T, *et al.* Development and validation of a patient-reported outcome measure to assess symptom burden after chimeric antigen receptor T-cell therapy. *Br J Haematol*. Published Online First: 2023. doi: 10.1111/BJH.18677

38 Wang XS, Srour SA, Whisenant M, *et al.* Patient-Reported Symptom and Functioning Status during the First 12 Months after Chimeric Antigen Receptor T Cell Therapy for Hematologic Malignancies. *Transplant Cell Ther*. 2021;27:930.e1-930.e10. doi: 10.1016/J.JTCT.2021.07.007

39 Zhang X, Lu XA, Yang J, *et al.* Efficacy and safety of anti-CD19 CAR T-cell therapy in 110 patients with B-cell acute lymphoblastic leukemia with high-risk features. *Blood Adv*. 2020;4:2325. doi: 10.1182/BLOODADVANCES.2020001466
